# Incidence of myocardial infarction in people with diabetes compared to those without diabetes: a systematic review protocol

**DOI:** 10.1186/s13643-022-01962-z

**Published:** 2022-05-12

**Authors:** Maria Narres, Tatjana Kvitkina, Heiner Claessen, Ellen Ubach, Georg Wolff, Maria-Inti Metzendorf, Bernd Richter, Andrea Icks

**Affiliations:** 1 Institute of Health Services Research and Health Economics, German Diabetes Center (DDZ), Leibniz Center for Diabetes Research at Heinrich-Heine-University Düsseldorf, Düsseldorf, Germany; 2 Institute of Health Services Research and Health Economics, Center for Health and Society, Medical Faculty, Heinrich-Heine-University Düsseldorf, Düsseldorf, Germany; 3 German Center for Diabetes Research (DZD), Neuherberg, Germany; 4 Division of Cardiology, Pulmonology and Vascular Medicine, Department of Internal Medicine, Medical Faculty, Heinrich-Heine-University Düsseldorf, Düsseldorf, Germany; 5 Cochrane Metabolic and Endocrine Disorders Group, Institute of General Practice, Medical Faculty, Heinrich-Heine-University Düsseldorf, Düsseldorf, Germany

**Keywords:** Myocardial infarction, Incidence, Diabetes, Population-based study, Systematic review

## Abstract

**Background:**

Diabetes mellitus is an established risk factor for acute myocardial infarction (AMI). Incidence of AMI in people with diabetes remains significantly higher than in those without diabetes. However, published data are conflicting, and previous reviews in this field have some limitations regarding the definitions of AMI and source population (general population or people with diabetes as a population at risk) and concerning the statistical presentation of results.

**Aims:**

To analyse the incidence of AMI in people with diabetes compared to those without diabetes and to investigate time trends.

**Methods:**

We will perform a systematic literature search in MEDLINE, Embase and LILACS designed by an experienced information scientist. Two review authors will independently screen the abstracts and full texts of all references on the basis of inclusion criteria regarding types of study, types of population and the main outcome. Data extraction and assessment of risk of bias will be undertaken by two review authors working independently. We will assess incidence rate or cumulative incidence and relative risk of AMI comparing populations with and without diabetes.

**Discussion:**

This review will summarise the available data concerning the incidence of AMI in people with and without diabetes and will thus contribute to the assessment and interpretation of the wide variations of incidence, relative risks and time trends of AMI in these populations.

**Systematic review registration:**

PROSPERO CRD42020145562

**Supplementary Information:**

The online version contains supplementary material available at 10.1186/s13643-022-01962-z.

## Introduction

Diabetes mellitus is an established risk factor for cardiovascular disease, including acute myocardial infarction (AMI) [1–3]. Most population-based studies have reported a substantially decreased time trend in AMI incidence in recent decades [4–7]. However, a recent US study analysing risk of AMI among people with diabetes showed that following a reduced risk of hospitalisation due to AMI in 1990–2010, the risk has since increased again in young and middle-aged people, while it has remained stable in older people (65 and over) [8]. Despite all improvements, the incidence of AMI in people with diabetes therefore remains up to four times higher than in those without diabetes [1, 5, 7, 9]. Furthermore, coronary heart disease remains a primary cause of death in people with diabetes [4, 10], and up to 40% of patients with AMI die before hospital admission [7]. Mortality following AMI in people with diabetes is high [11], and a recent systematic review did not find any positive temporal change concerning mortality risk after AMI among people with diabetes compared to those without diabetes [12]. Moreover, high costs incurred due to AMI have a significant impact on total medical costs in people with diabetes [13, 14].

Thus, the incidence of AMI in the population with diabetes nowadays still remains an important indicator of diabetes care. Nevertheless, papers analysing the incidence of AMI showed wide variations in incidences, and it is not clear whether these differences are associated with diabetes care or could at least partially be explained by methodological discrepancies between studies regarding study population, definition of AMI, recording of AMI, definition of diabetes and statistical presentation of results. Previous reviews investigated diabetes mellitus as a risk factor for cardiovascular disease or coronary heart disease [3, 15–17], but no systematic review has been conducted with a focus on incidence of myocardial infarction in people with diabetes compared to those without diabetes based on population-based studies. The aim of this systematic review is therefore to (a) analyse the incidence of myocardial infarction in people with diabetes compared to those without diabetes, (b) describe the discrepancies in incidence of myocardial infarction regarding age, sex, ethnicity and geographic region and (c) estimate time trends.

## Materials and methods

The proposed review protocol adheres to the PRISMA-P guideline [18].

### Eligibility criteria

#### Types of studies

All population-based studies analysing incidence rates in people with diabetes compared to those without diabetes using both prospective and retrospective designs will be included in this review.

#### Study population

A. The study population shall be defined using official statistics, for instance citizens of a country or inhabitants of a defined administrative region or all those insured by a statutory health insurance provider.
B. The population with diabetes shall be precisely described (register, survey data, estimation based on age-sex-specific prevalence data). Incidence can be reported in people with type 1 diabetes, type 2 diabetes or without a distinguished diabetes type. We will also consider previously used diabetes classifications, namely insulin-dependent (IDDM) and non-insulin-dependent diabetes mellitus (NIDDM). The population without diabetes will be considered only for the purpose of comparison of incidences among people with diabetes.

#### Outcomes

The main outcome of the included studies should be the incidence of AMI in people with and without diabetes. Both non-fatal and fatal AMI should be recorded.

#### Epidemiological measures

Incidence rate (IR) or cumulative incidence (CumI): To ensure the appropriate comparison between people with diabetes to those without, incidence should be presented at least as age-(sex)-adjusted or standardised incidence. We shall also investigate risk ratios (RR), comparing the incidence of AMI among people with and without diabetes. The attributable risk (*AR* = proportion of AMI risk among people with diabetes that is attributable to diabetes) and the population attributable risk (*PAR* = proportion of AMI risk in the whole population that is attributable to diabetes) shall be considered where available.

#### Information sources

We will perform a systematic literature search in the MEDLINE, Embase, and LILACS literature databases. This database selection adheres to recommendations on searching for epidemiological studies [19]. Moreover, we will aim to identify potentially eligible studies by using additional methods, such as checking reference lists of review articles and relevant studies.

#### Search strategies

To fulfil the requirements for conducting systematic reviews according to the established guidelines for meta-analyses of observational studies in epidemiology (the MOOSE group [20]), a comprehensive systematic search strategy was designed by an experienced information scientist and tested against eight known relevant references from previous systematic reviews. All search strategies are available in the Additional file 2: Appendix.

All database records yielded by the search will be exported into EndNote and duplicates removed.

#### Study selection process — inclusion and exclusion criteria

Two authors will independently screen the titles and abstracts of all references to identify original research of the incidence of AMI according to our inclusion criteria. Subsequently, two review authors will independently screen the full-text articles of abstracts identified in this initial phase. Potential disagreements regarding the inclusion or exclusion of studies shall be solved in discussion with a third review author.

Original full-text articles will be included if they fulfil the criteria concerning types of study (population-based longitudinal studies only), types of population (with and without diabetes) and main outcome (fatal and non-fatal AMI), regardless of the time period and year of publication of the study, type of diabetes, age and sex distribution or ethnicity. Since we expect studies with largely varying study designs and data sources, we will not prespecify a definition of AMI. However, we will precisely describe the definitions.

Studies will be excluded if (a) they only reported incidence of non-fatal AMI; (b) they solely report incidences of myocardial infarction among people with diabetes, without comparison to people without diabetes; (c) incidence rates are reported in relation to the total (with and without diabetes) population and do not exclusively use the population with diabetes as an at-risk population; (d) only crude incidence rates (calculated by dividing the total number of cases in a given time period by the total number of persons in the population) are reported; and (e) studies are published in a language other than English.

### Data quality

Two authors will independently perform a critical appraisal of studies to evaluate methodological quality and potential risk of bias in the eligible studies using the modified checklist adapted to the Methodological Evaluation of Observation Research (MORE), Scottish Intercollegiate Guidelines Network (SIGN) and the Cochrane Consumers and Communication Review Group’s study quality guide. Using this checklist, we will assess features that could potentially bias estimates for myocardial infarction and will rank potential sources of bias as low, high or unclear risk according to the Cochrane recommendations [21].

#### Data extraction and synthesis

We will first develop a data extraction sheet based on the Cochrane Consumers and Communication Review Group’s data extraction template before carrying out a pilot test using five randomly selected included papers and then refine the data extraction sheet accordingly. One review author will extract data (see data items below) from included articles; the other author will check the extracted data. Both authors will resolve disagreements by discussion. If no agreement can be reached, a third author shall be called upon to make a decision.

We will extract the following information from each included article: first author, publication year, country, study period, study population, characteristic of people with diabetes, definition of fatal and non-fatal AMI, the presence or absence of comorbidity that is relevant for AMI incidence (e.g. previous AMI or CHD or CVD) in people at risk, absolute numbers and incidence (incidence rate or cumulative incidence) of AMI, relative risk (comparing the incidence of AMI in people with and without diabetes) and a time trend where available. We will recalculate the reported IR per 100,000 person-years, if originally not reported as such.

Previous experience with similar reviews [22, 23] suggests that studies will be too heterogeneous to allow for a quantitative summary of results. Hence, data will be presented descriptively in tables. The results from included studies will be presented as age-sex-adjusted or standardised estimates. All estimates will be presented with 95% confidence intervals (95% CI), if available.

## Discussion

It is well known that diabetes mellitus is a risk factor for AMI. However, studies analysing the incidence of AMI in people with diabetes show marked variations in incidence, relative risks and time trends of AMI, partly due to differences in the identification of the study population, definition of AMI, diabetes and the statistical presentation of results. The aim of our systematic review is to analyse the incidence of AMI in people with diabetes compared to those without diabetes and to investigate time trends based on population-based studies. The strength of this review is a systematic search approach that will be based on clearly determined search strategies. Moreover, the selection and the critical appraisal of studies will be performed by two researchers using predefined inclusion and exclusion criteria. A potential limitation of this review lies in the heterogeneity of the included cohort studies.

In summary, the results of the current systematic review will contribute to the assessment and interpretation of the wide variations of incidence and time trends of AMI in people with and without diabetes. This information will be of benefit to clinicians, researchers and healthcare decision-makers.

## Supplementary Information

**Additional file 1: Appendix 1.** PRISMA-P Checklist.

**Additional file 1: Appendix 2.** Search strategies.

## Data Availability

The datasets generated during and/or analysed during the current study are available from the corresponding author on reasonable request.
